# Single-shot memory-effect video

**DOI:** 10.1038/s41598-018-31697-8

**Published:** 2018-09-07

**Authors:** Xiaohan Li, Andrew Stevens, Joel A. Greenberg, Michael E. Gehm

**Affiliations:** Department of Electrical and Computer Engineering, Duke University, Box 90291, Durham, NC 27708 United States

**Keywords:** Optical sensors, Imaging and sensing

## Abstract

Imaging through opaque scattering media is critically important in applications ranging from biological and astronomical imaging to metrology and security. While the random process of scattering in turbid media produces scattered light that appears uninformative to the human eye, a wealth of information is contained in the signal and can be recovered using computational post-processing techniques. Recent studies have shown that statistical correlations present in the scattered light, known as ‘memory effects’, allow for diffraction-limited imaging through opaque media without detailed knowledge of (or access to) the source or scatterer. However, previous methods require that the object and/or scatterer be static during the measurement. We overcome this limitation by combining traditional memory effect imaging with coded-aperture-based computational imaging techniques, which enables us to realize for the first time single-shot video of arbitrary dynamic scenes through dynamic, opaque media. This has important implications for a wide range of real-world imaging scenarios.

## Introduction

Conventional optical imaging techniques create a one-to-one mapping between object and image planes. This approach assumes that one can measure ballistic, or non-scattered, light from an object. When a sufficiently opaque material intervenes, most of the light instead undergoes scattering and yields a random speckle pattern at the detector, rendering isomorphic imaging impossible. Nevertheless, a variety of techniques have been developed to allow imaging through opaque materials by filtering out the scattered light^1–3^, performing wavefront shaping of the light incident on the scatterer^4–6^, conducting detailed statistical modeling of the scatterer^7^, or exploiting intrinsic correlations in the scattered light^8–11^. Of these approaches, only the latter method, known as ‘memory effect’ (ME) imaging^12,13^, allows for imaging through highly scattering media without the need for detailed knowledge of or access to the scatterer, object, or illumination. However, all these previously-demonstrated techniques require that the scatterer and/or object remain stationary during the measurement, which fundamentally limits their applicability.

Several recent approaches have attempted to relax the requirement that the object and/or scatterer remain stationary. For example, imposing a temporal modulation on the source^14^ or having direct access to the system point spread function (PSF)^15^ enables fast imaging through quasi-static scattering media. Relatedly, Cua *et al*.^16^ show that proper filtering in correlation space enables one to recover the shape of an unchanging object undergoing simple (e.g. linear translational) motion. Finally, Edrei *et al*.^17^ use the so-called shower curtain effect to image a static object through a dynamic scatterer. However, this method requires knowledge about the location of the scatterer as well as significant signal averaging, which does not allow for a direct generalization to moving objects. Thus, none of the previously-developed methods can faithfully image arbitrary object motion through a dynamic, unknown scatterer.

Here, we demonstrate a method for imaging through opaque media when the object and scatterer involve arbitrary dynamics that may even exceed the measurement rate of the detector. By using a modulator to temporally-code the speckle image on the time scale of the scene dynamics^18^ (i.e., faster than the detector frame rate), we ‘timestamp’ the dynamics in the time-integrated signal collected by the detector. We then use a dictionary learning approach^19^ to recover multiple high-speed speckle frames from a single acquisition, and independently process these de-multiplexed speckle images to estimate the scene at each frame, yielding an *effective framerate* that is faster than the detector. In this way, we realize single-shot video through an opaque scatterer. The technique makes no assumptions about the dynamics of the object or scatterer and requires modifications only to the detector system (i.e., does not require access to the source or scatterer). Furthermore, the method is independent of the post-processing performed on the recovered speckle, and therefore provides an effective boost to the frame rate of any previously-studied coherent imaging technique and broadens their efficacy in real-world scenarios.

More generally, our results show that low-contrast speckle can alternatively be thought of multiple, high-contrast speckle fields that have been *multiplexed* together. Our work can therefore be viewed as an extension of coded aperture compressive temporal imaging^18^ (CACTI) to non-natural images (even for something as naively non-compressible as speckle). As a result of this mathematical similarity, variants of the physical and algorithmic tools developed in the fields of computational and compressive imaging can be brought to bear to code the individual channels and then demultiplex them post detection.

## Results

### Measurement technique

**Figure 1:**
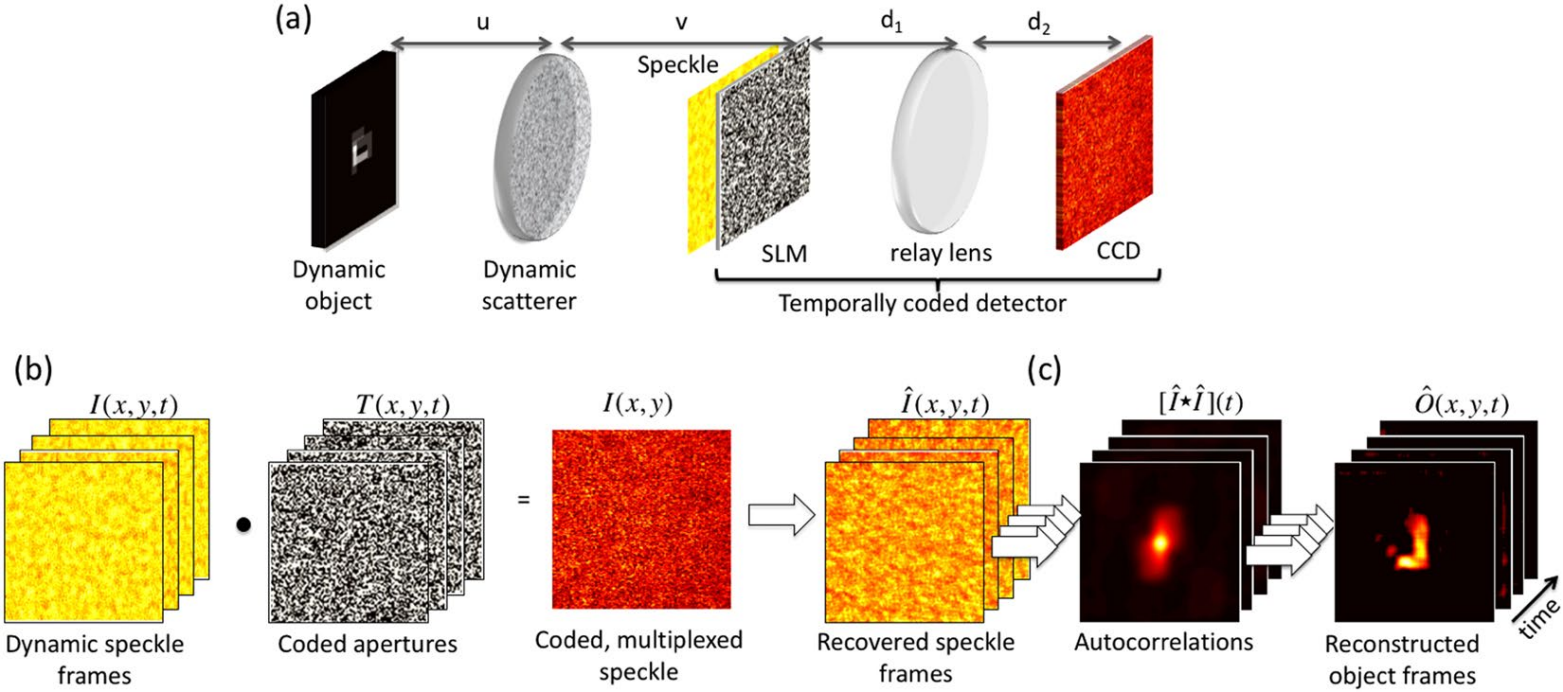
Schematic and conceptual description of coded single-shot video through an opaque medium. (**a**) Spatially incoherent light from an object (static or dynamic) propagates a distance *u* and passes through a diffuser (static or dynamic). Propagation an additional distance *v* produces a speckle pattern at the plane of a dynamically reconfigurable SLM, which imposes a spatio-temporally varying intensity modulation. A relay lens images the SLM plane onto a CCD camera. Together, the SLM, relay lens and CCD correspond to our temporally coded detector setup. (**b**,**c**) Measurement structure and data processing chain for speckle demultiplexing and correlation-based image recovery, respectively. Each speckle frame $I(x,y,t)$ is multiplied by a distinct coded aperture $T(x,y,t)$ and the resulting coded speckle patterns are summed into a single measurement at the CCD $I(x,y)$ (the dot in the figure denotes the Hadamard product). We recover independent speckle frames $\hat{I}(x,y,t)$ using a dictionary learning algorithm, calculate the autocorrelation of each frame separately, and reconstruct the time-dependent object $\hat{O}(x,y,t)$ by applying an iterative phase retrieval algorithm (see Supplemental Materials Sec. 4 for more details).

Figure 1 shows a schematic of the experimental configuration as well as the associated coding and image recovery strategy. We consider a dynamic object (whose angular extent fits within the ME field of view, FOV^8,9,15^) located a distance *u* behind an opaque scatterer (see. Fig. 1a and Supplemental Materials Fig. 1). Light from the object passes through a dynamic scatterer and generates a time-varying speckle pattern in the far field *I(x,y,t)*. In a traditional ME imaging setup, one places the detector a distance *v* behind the scatterer to record the speckle; in our coded aperture configuration, we instead place a second SLM at this plane to spatio-temporally modulate the speckle. While a variety of code patterns are possible, we use a sequence of random binary patterns *T(x,y,t)* to minimize temporal correlations between the codes. We then image the coded speckle onto the detector plane. This single, low-contrast coded speckle image *I(x,y)* represents the superposition of the coded speckle patterns reaching the detector over the course of the acquisition time, and can be described as the Hadamard product of the speckle and the coded aperture pattern (i.e., pixelwise multiplication summed over time frames, Fig. 1b).

While such low-contrast speckle is typically considered to be ‘washed out’ or otherwise devoid of information^20^, we instead view the incoherent sum of many speckle images as a form of multiplexing. To recover separate, time-ordered speckle frames $\hat{I}(x,y,t)$ from the single, multiplexed speckle measurement, we use a compressed sensing (CS) algorithm with an imposed sparsity prior^21^. However, speckle is not a natural image and is not necessarily sparse in a wavelet basis^22^; instead, we use a patch-based dictionary-learning approach^19^ to determine a general sparse representation of speckle (see Methods section below and Supplemental Materials Sec. 3 for details). We note that a sparse representation via a learned dictionary is possible, as speckle is not truly random and contains spatial correlations on the scale of the grain size when the grain is oversampled by the detector. At the same time that the CS algorithm de-multiplexes the separate temporal channels, the algorithm also performs denoising and inpaints the parts of each speckle image blocked by the coded aperture. By making this multiplexed measurement in the presence of detector noise, we can potentially outperform a system that makes separate, higher-frame-rate acquisitions via the multiplex advantage^23^.

**Figure 2:**
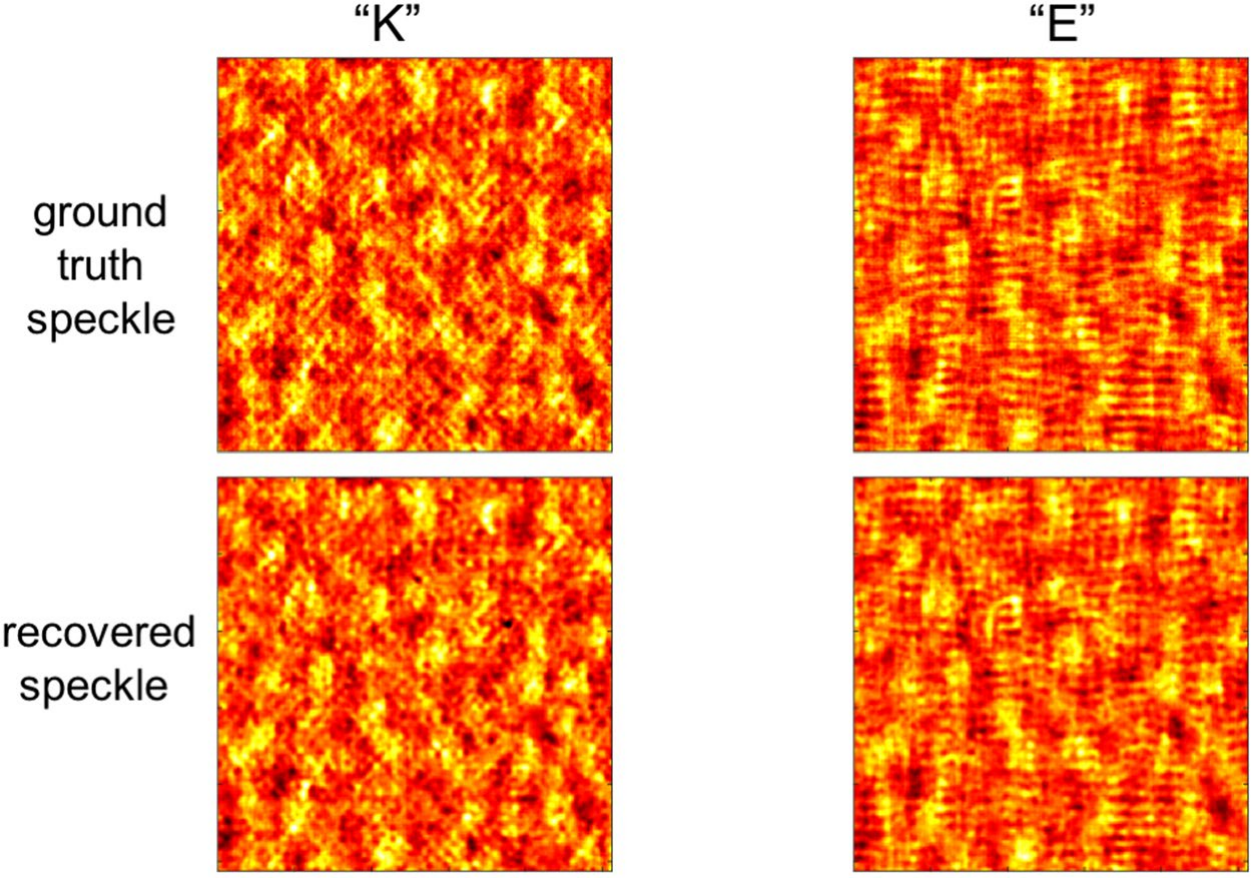
Ground truth and recovered speckle subframes. (Top row) Separate speckle images obtained using separate measurements of static objects (here we use the letters ‘K’, ‘E’, respectively, as shown in Fig. 3). (Bottom row) Speckle images recovered from a single, coded speckle measurement including dynamic sum of the same. The normalized correlation between ground truth and recovered speckle for ‘K’ and ‘E’ are 0.915 and 0.928.

**Figure 3:**
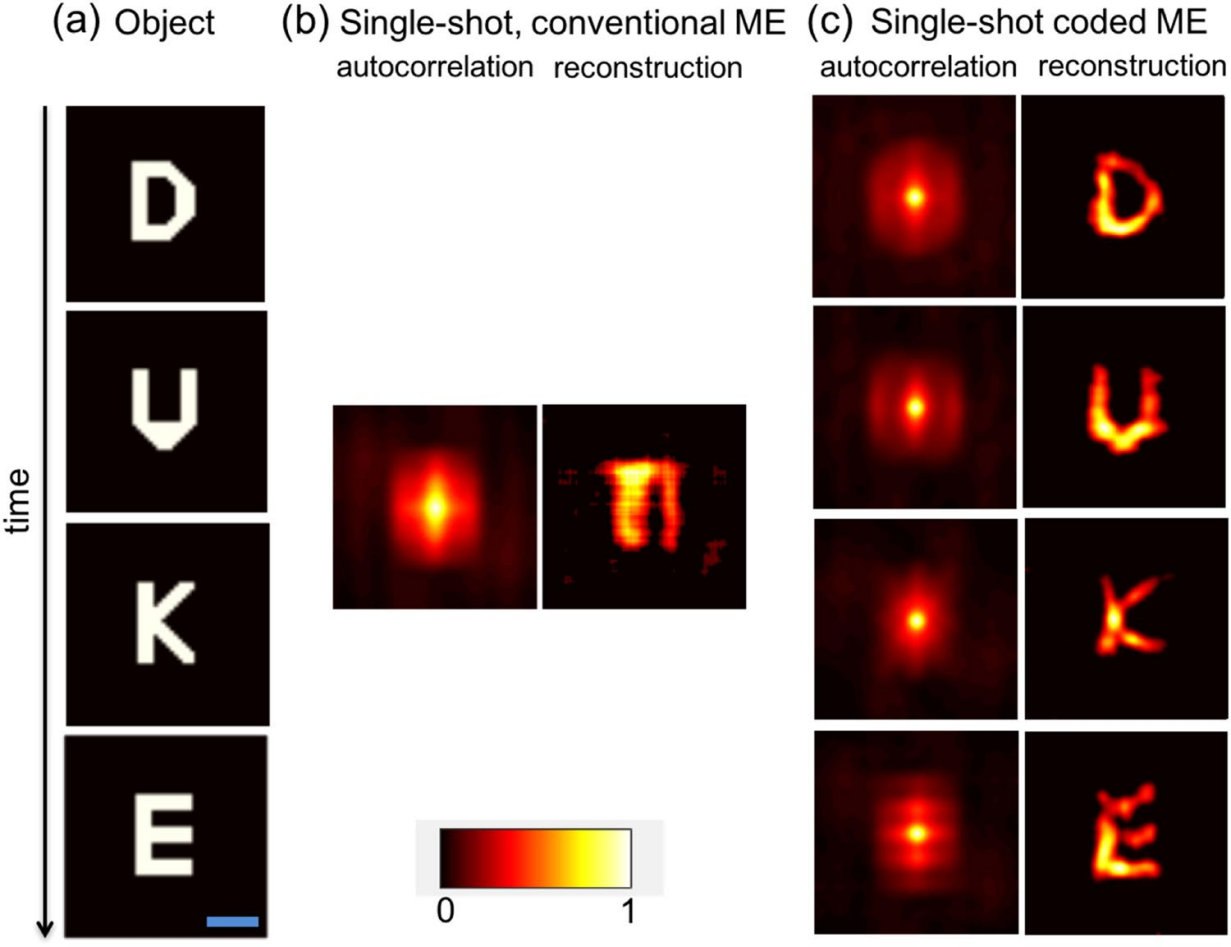
Experimental demonstration of imaging a dynamic scene through a static scatterer. (**a**) Ground truth object frames (time proceeds from top to bottom, as indicated by the arrow). The extent of each letter is 576 × 720 μm (corresponding to an angular field of view of 0.22 × 0.28 degrees), and the blue scale bar indicates 500 μm. (**b**,**c**) Speckle autocorrelation(s) and reconstructed object frame(s) at each time obtained via a single measurement using conventional ME imaging and a single measurement using our coded aperture ME imaging. For all measurements, the diffuser is stationary. The color bar shows the image intensity, which is normalized to the range [0,1].

Figure 2 shows representative speckle patterns produced in our system for input objects corresponding to the letters “K” and “E” (as shown in Fig. 3). We determine the ground truth speckle pattern for a given object by removing the coded aperture and measuring the scattered light in the absence of the object or scatterer motion (see Fig. 2 top row). These speckle images can then be compared to the recovered (i.e., de-multiplexed) speckle patterns obtained from a single, coded measurement (i.e., for *N*_*t*_ = 2, see Fig. 2 bottom row). We find that our technique allows us to accurately recover these highly-complex speckle images (see Supplemental Materials Fig. 5 for more details), and does not require any assumptions regarding temporal correlations in the speckle from one frame to the next (e.g., stemming from either the object or scatterer motion). While such prior knowledge could further improve system performance^24^, such an assumption fundamentally limits the range of motion to within the ME FOV^16^. In contrast, our coding technique is capable of imaging small objects (i.e. with an angular extent less than the ME FOV) with arbitrary temporal evolution as they move through regions exceeding the ME angular FOV.

Once we recover the ${N}_{t}$ separate speckle frames, we process each frame individually using traditional ME correlation-based processing^8,9^. Namely, we first calculate the autocorrelation of the speckle pattern $[\hat{I}\ast \hat{I}]\,(t)$, which is directly related to the autocorrelation of the object. We then use a phase retrieval algorithm^25^ to estimate the object $\hat{O}(x,y,t)$ at each time, and concatenate the resulting images to create a video of the dynamic scene that is robust against arbitrary motion of the object and/or scatterer. While the use of phase retrieval ultimately renders ME imaging most applicable to imaging bright objects on a dark background, the reduction of motion blur afforded by our technique reduces the effective object complexity^9^ and thus improves the resulting image contrast in the presence of motion (see Supplemental Materials Sec. 3e).

### Experimental demonstration of snapshot ME video

As a first example of our technique, we consider a static diffuser and a dynamic object consisting of a time-ordered sequence of the letters ‘D’, ‘U’, ‘K’, and ‘E’ (see Fig. 3a). When we use conventional, single-shot ME imaging (i.e., for *T*(*x*, *y*, *t*) = 1), the speckle autocorrelation lacks distinct structure and the single estimated image is obviously incorrect (see Fig. 3b). Unlike in the case of conventional motion blur, the resulting image is not simply the linear sum of the underlying objects. This is due to the fact that the PSF is distributed (resulting in nonlocal image distortions) and that the autocorrelation operator is not distributive over addition (i.e., the autocorrelation of the sum of two signals is not the sum of their respective autocorrelations). As a result, the image quality can degrade significantly due to even slight dynamics.

In contrast, our single-shot coded ME scheme allows us to recover separate, time-resolved autocorrelations that faithfully reveal the object dynamics (see Fig. 3c). This result demonstrates that our method works for object dynamics lacking a priori temporal correlations, and is valid for arbitrary absolute time scales as long as the object and code dynamics are well-matched and faster than the detector acquisition time.

**Figure 4:**
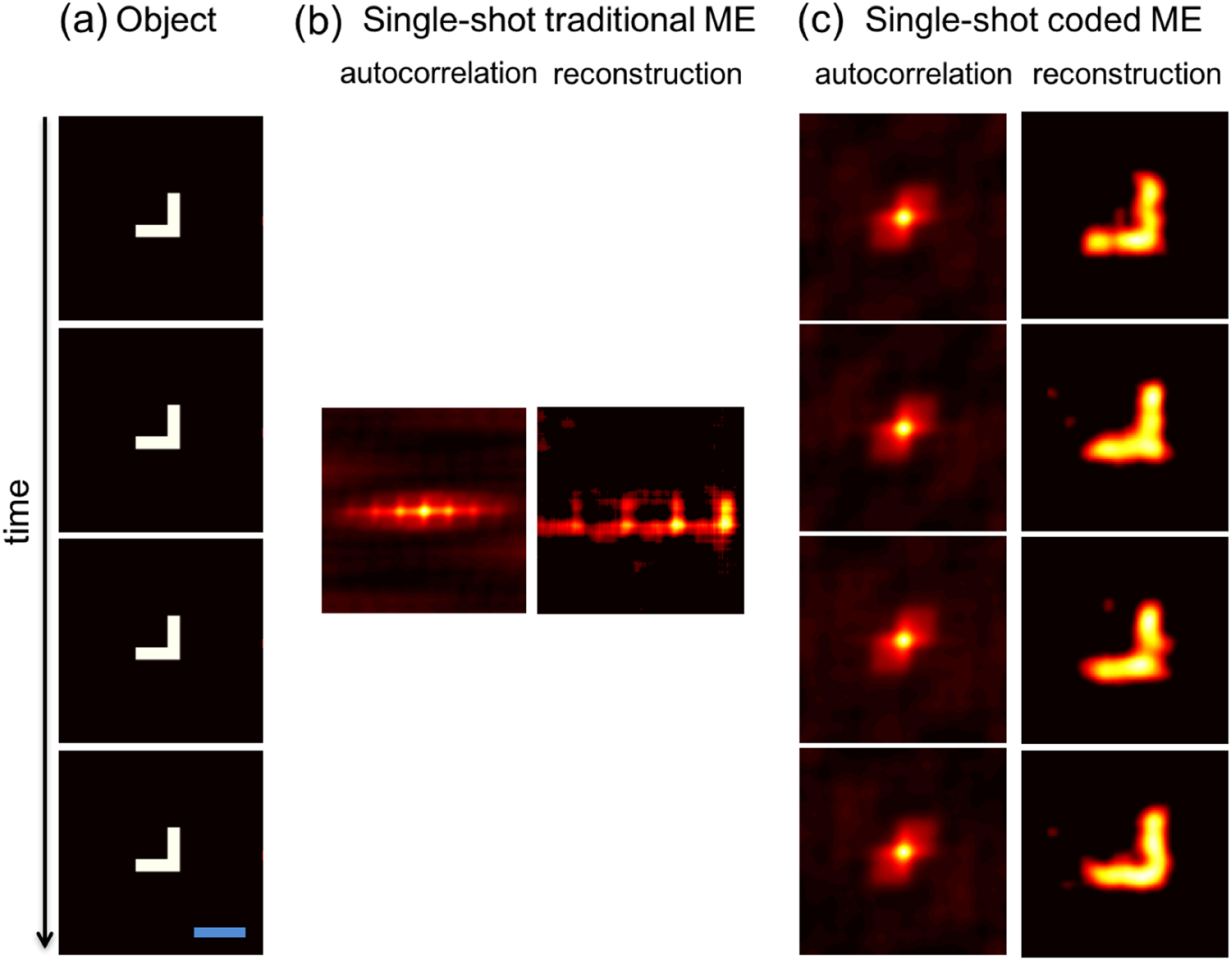
Experimental demonstration of imaging a static scene through a dynamic scatterer. (**a**) Ground truth object (angular field of view is 0.19 × 0.19 degrees). (**b**,**c**) Speckle autocorrelation(s) and reconstructed object frame(s) at each time obtained via a single measurement using conventional ME imaging and a single measurement using our coded aperture ME imaging, respectively. The average correlation amongst the four reconstructed object frames via the coded ME approach 0.88. For all measurements, the object is stationary.

We next consider a stationary object and dynamic scatterer, which represents a well-known hardware approach to ‘eliminate’ speckle. We linearly translate the diffuser in small (i.e., within the ME range), discrete steps during the acquisition to make clear the impact of the dynamics. For the image shown in Fig. 4a), the traditional single-shot ME approach produces an autocorrelation and recovered image that is blurred according to the diffuser motion (see Fig. 4b). However, our single-shot coded ME technique is robust to this motion and enables us to recover multiple, time-resolved speckle images that lead to unblurred images of the object at each time (see Fig. 4c). The static object is correctly observed as such, which demonstrates that our technique is robust against diffuser dynamics. We note that, while this example is limited to motion within the ME range, the approach and general results still hold in the case of random and/or continuous motion as long as the dynamics within any sub-frame remain instantaneously within the memory effect range (see Supplemental Materials Figs 7 and 8).

**Figure 5:**
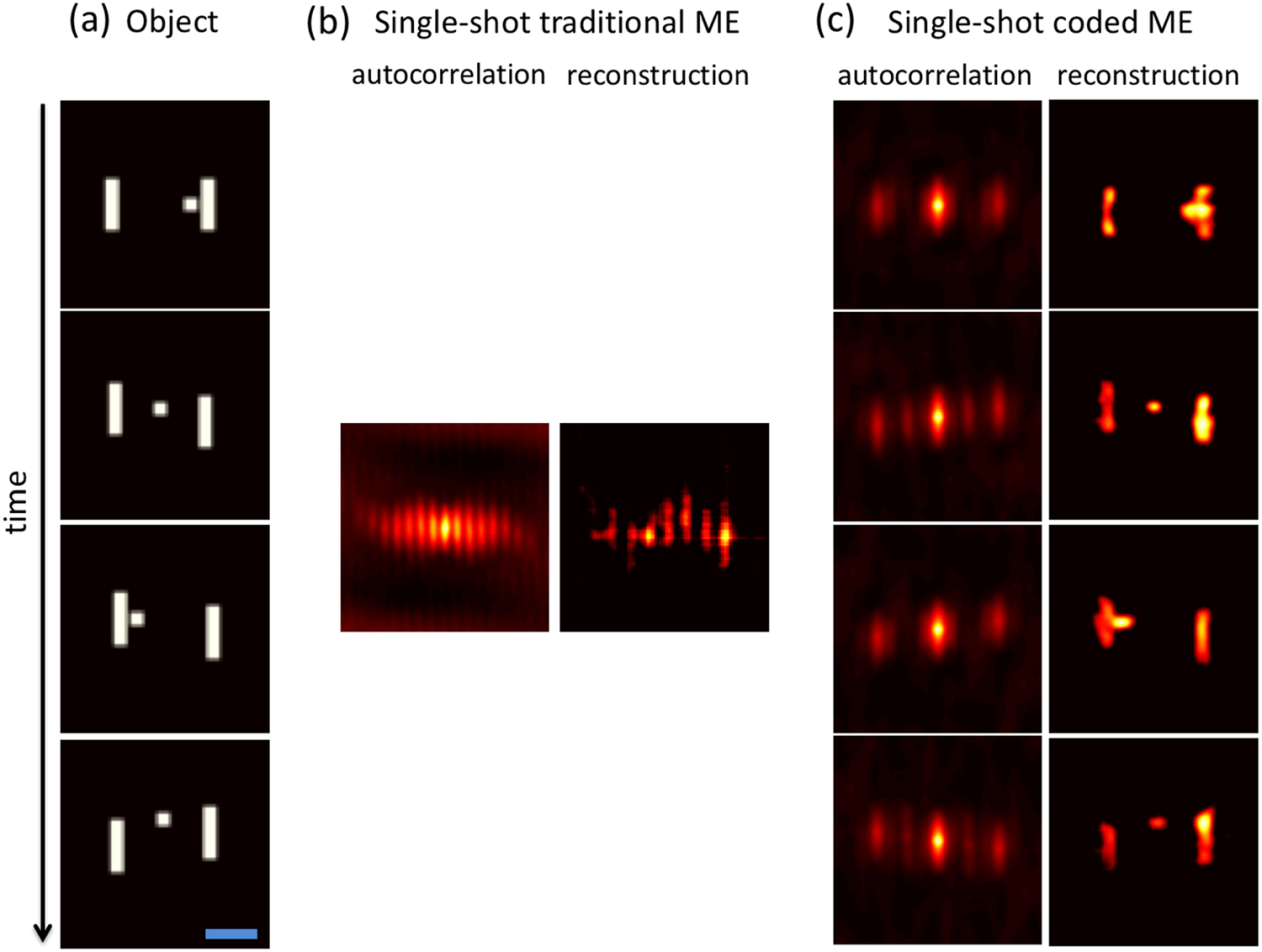
Experimental demonstration of imaging a dynamic scene through a dynamic scatterer. (**a**) Dynamic ground truth object (angular field of view is 0.44 × 0.22 degrees). (**b**,**c**) Speckle autocorrelation(s) and reconstructed object frame(s) at each time obtained via a single measurement using conventional ME imaging and a single measurement using our coded aperture ME imaging, respectively.

As a final example, we consider a dynamic object imaged through a dynamic scatterer. We again make no assumptions about the dynamics and take simulated frames of a paddle-and-ball video game as our dynamic scene (see Fig. 5a). We again use discrete, linear translation motion for our diffuser. As expected, the single-shot conventional ME image quality is poor, and the approach fails to provide meaningful information about the object dynamics (see Fig. 5b). The single-shot coded ME approach, however, enables a clear understanding of the evolution of the scene (see Fig. 5c). Furthermore, the results are largely unaffected by the motion of the scatterer, as can be seen by comparing them against the ground truth object.

## Discussion

For the results shown above, we typically set the camera integration time to approximately 100 s in order to overcome the readout detector noise on the CCD camera and achieve an excellent signal to noise ratio (SNR, typically 100). Therefore, the acquisition speed in our experiment is limited by the light source rather than the SLM (coded aperture) refresh rate. With sufficiently bright sources where the SLM refresh rate acts as a bottleneck, one can simply switch to an alternative coding approach (e.g., using DMD or a physical coded aperture that is translated and/or rotated rapidly). The technique does not require this measurement duration or SNR, though, and we find that the performance remains fairly uniform down to camera frame rates of approximately 1 Hz (i.e., recovered sub-frame rates of N_t_ Hz), at which point the image quality begins to decrease gracefully. We note, however, that the image quality obtained when using a conventional ME imaging approach with our system (i.e., for a static object and scatterer and no coded aperture) also begins to decrease for the same parameters as our coded scheme, indicating that our approach does not significantly increase the SNR required for imaging. Through the use of alternate sources, configurations, and/or detectors, others have demonstrated conventional ME imaging with integration times as short as 10 ms^9^, which implies that our technique can be extended to much faster absolute frame rates (i.e. potentially realizing frame rates of 100*N_t_ Hz). Thus, the specific times used in this experiment do not represent a fundamental limit; rather, they demonstrate the potential to realize improved performance relative to conventional ME imaging, and give excellent results for our particular system.

As has been discussed elsewhere, though, fundamental tradeoffs exist between the achievable spatial and temporal resolution in these types of temporally coded systems^26,27^. While a complete analysis of this dependence is beyond the scope of our current study, our preliminary analysis shows that the recovered speckle fidelity can drop considerably (e.g., the correlation between recovered and ground truth speckle can be as low as 75%) before significant degradation of the resulting ME video is observed (see Supplemental Materials Fig. 5). To further improve the accuracy of the resulting ME video and potentially make it more robust to imperfect recovery of the associated speckle subframes, alternative approaches to the image recovery may be used.^28,29^

In summary, we have demonstrated a new computational imaging architecture that combines physical layer coding with memory effect imaging to realize single-shot video through a dynamic, opaque scatterer. The technique does not require access to the object or scatterer and applies to light that either undergoes scattering via transmission through an opaque medium or via reflection from a rough surface. This opens avenues for imaging in rapidly-changing turbid media, such as due to atmospheric motion in astronomical speckle interferometry^30^ or for *in vivo* imaging of biological samples^31,32^. In addition, it creates possibilities for recording dynamic scenes under conditions in which direct visibility is precluded. More generally, this marriage of correlation-based imaging with state-of-the-art compressed sensing modalities can be expanded to other degrees of freedom (e.g., spectral or polarization^33–35^) to enable high-dimensional imaging of previously-inaccessible phenomena.

## Methods

### Experimental setup

The complete experimental setup is presented in Supplemental Materials Fig. 1. A 250 mW Opnext laser diode (Thorlabs HL6388MG) operating at 640 nm illuminates an integrating sphere (Thorlabs IS236A-4). The temporally coherent but spatially incoherent light from the output port passes through a SLM (HOLOEYE LC2012, 36 μm pixel pitch), which is located between a pair of crossed polarizers and used to generate the dynamic object. A 600 grit ground glass diffuser (Thorlabs DG20–600-MD) is mounted on a translation stage (Thorlabs MF A-CC) 300 mm from the SLM. A 4.5 mm diameter aperture immediately after the diffuser limits the scattered light that passes through a beam splitter (BS, Thorlabs CCM1-PBS251/M), located 12 mm away. The transmitted light passes through a quarter wave plate (QWP, Thorlabs WPQ20ME-633), reflects off the coding SLM (HOLOEYE Pluto Phase Only SLM, 8 μm pixel pitch), passes back through QWP, and reflects off the BS. An achromatic triplet (Thorlabs TRS254-040-A-ML, f = 40.6 mm) images the SLM plane onto a camera (SBIG STT-3200). The camera consists of 1472 × 2184 pixels (with a pitch of 6.8 μm) and was operated with an integration time of 600 to 1200 s. To minimize background light and stray reflections, we implement a series of bellows between the optical elements and covered the setup with a black box.

### Speckle recovery

We use beta process factor analysis (BPFA^19^) to learn an overcomplete dictionary from a set of training data. The training data consists of 18 different 1024 × 1024 representative speckle images, split into 16 × 16 pixel patches (i.e. a total of 73728 training sets). The dictionary only needs to be trained once off-line and works excellently for various geometric arrangements of the system and different diffusers. Given this dictionary, we used a matrix inverse-update implementation^36^ of the orthogonal matching pursuit (OMP) algorithm^37^ which projects the multiplexed speckle to the dictionary space and iteratively calculates the optimal dictionary elements and corresponding coefficients to recover the subframe speckle patterns. (see Supplemental Material Sec. 3 for additional details).

### Speckle pre-processing

We first normalize the speckle image by dividing it by a low pass version of itself (obtained by convolving the raw image with a uniform 220 × 220 matrix). We then choose a 752 × 752 pixel patch of the normalized image (limited by image aberrations in the relay optics), smooth it using a Gaussian filter with a standard deviation of 1 pixel, and apply a Tukey window^38^ with r = 0.1 (where 0 < r < 1 describes the filter falloff near the edges) to avoid any edge effects. Finally, we calculate the autocorrelation of the processed speckle.

### Phase retrieval

The phase retrieval algorithm was implemented using the conjugate gradient descent algorithm (see Supplemental Materials Sec. 4). We use a random initial guess for the object and estimate the magnitude of the Fourier transform of the object by taking square root of the Fourier transform of the product of autocorrelation and a 256 × 256 pixel 2D Tukey window. We enforce realness and non-negativity constraints on the object and typically run 3600 iterations. Once the image is recovered, we threshold values smaller than ten percent of the maximum value to zero in order to reduce apparent background noise, consistent with the practice in other memory-effect publications such as ref.^9^. No filtering or other image processing is applied.

## Electronic supplementary material

Supplementary Information

## Data Availability

The datasets generated during and/or analyzed during the current study are available from the corresponding author on reasonable request.
