# Supplementary material for: Single-shot memory-effect video
Source: Sci Rep. 2018 Sep 7;8:13402. doi: 10.1038/s41598-018-31697-8 (PMC6128846; doi:10.1038/s41598-018-31697-8)
Supplement: Supplementary file 1 — Supplementary Information [file 41598_2018_31697_MOESM1_ESM.pdf]

Supplementary Information for

Single-shot memory-effect video

Xiaohan Li, Andrew Stevens, Joel A. Greenberg, Michael E. Gehm

Department of Electrical and Computer Engineering, Duke University, Box 90291, Durham, NC, 27708

1. Experimental setup

The experimental setup consists of three different sections: 1) a bright, dynamic object, 2) a dynamic scatterer, and 3) a coded detector (see Supplemental Figure 1). To create the bright object, a 250 mW Opnext laser diode (Thorlabs HL6388MG) operating at 640 nm illuminates an integrating sphere (Thorlabs IS236A-4). The integrating sphere ensures spatial incoherence of the illumination but, more importantly, it creates a largely static distribution of light such that the observed speckle at the detector does not change over the time scale of the experiment. This is critical for measuring the *ground truth* (i.e., un-multiplexed, un-coded) speckle with which to determine the fidelity of the recovered speckle subframes. The temporally coherent but spatially incoherent light from the output port passes through a SLM (HOLOEYE LC2012, 36 μm pixel pitch), which is located between a pair of crossed polarizers and used to generate the dynamic object.

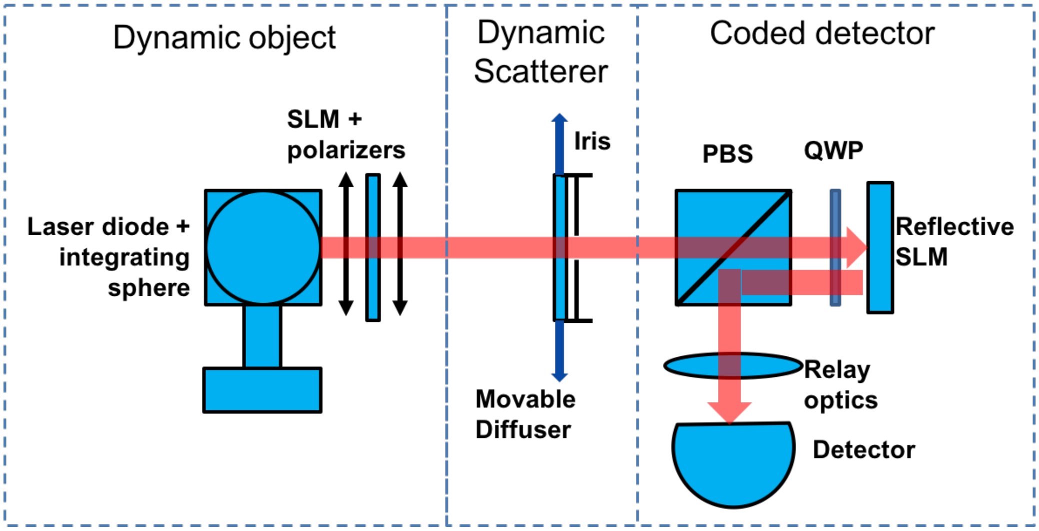

Supplemental Figure 1 | Schematic of experimental optical setup: The dynamic object is created by a transmissive SLM illuminated by narrowband laser light exiting an integration sphere. The dynamic scatter is created by mounting a ground glass diffuser and iris on a linear translation stage. The coded detector consists of a reflective SLM for implementing intensity modulations, relay optics, and a CCD camera.

The dynamic scatterer, which consists of a 600-grit ground glass diffuser (Thorlabs DG20-600-MD), is mounted on a translation stage (Thorlabs MF A-CC) u=300 mm from the SLM. A 4.5 mm diameter aperture immediately after the diffuser limits the scattered light that passes through a

beam splitter (BS, Thorlabs CCM1-PBS251/M), located v=12 mm away. The iris aperture is chosen to balance throughput and speckle contrast but does not, as in a typical lens-based imaging system, determine the system's field of view (FOV). Instead, the FOV is determined by ME angular range, since the motion and/or extent of the object in any temporal subframe must fit within the ME angular range. We note, though, that our technique is agnostic to whether the scatterer is in transmission or reflection mode, as long as it is sufficiently random and satisfies the requirements for producing an angular memory effect.

The scattered light then passes through a quarter wave plate (QWP, Thorlabs WPQ20ME-633), reflects off the coding SLM (HOLOEYE Pluto Phase Only SLM, 8 μm pixel pitch), passes back through QWP, and reflects off the BS. An achromatic triplet (Thorlabs TRS254-040-A-ML, f=40.6 mm) images the SLM plane onto a camera (SBIG STT-3200). The camera consists of 1472 x 2184 pixels (with a pitch of 6.8 μm). To minimize background light and stray reflections, we implement a series of bellows between the optical elements and covered the setup with a black box.

The code pattern that we use in the experiment is a binary, random pattern with a 50% duty cycle (i.e., half of the available area is fully reflective) as a compromise between sufficient coding (to make each temporal sub-frame sufficiently unique from the others) and maximizing the measurement signal to noise ratio (SNR). The code feature size should be roughly equal to or smaller than the average speckle size (see Supplemental Material Sec. 3 g), although smaller code features give improved performance as they effectively increase the code uniqueness. While the ideal configuration would have pixel-level coding at the detector plane, the constraints imposed by the available components require us to code in one plane and separately image the coding plane at the detector. Due to the finite resolution of the relay optics and the slight mismatch between the SLM and detector pixel sizes, we use code features that are 1 x 2 pixels on the SLM and employ a magnification of $M = d_2/d_1$ =2.08 between the coding SLM and detector planes. This makes each code feature correspond to 2.4 x 4.8 detector pixels to ensure sufficient sampling of the code pattern.

In a typical experiment, we use discrete, deterministic, synchronized motion of the object, scatterer, and coded aperture. In addition, we measure the speckle with and without coding as well as with all elements stationary (i.e., separate measurements of each temporal subframe). This allows us to directly compare the speckle produced by the conventional (i.e., un-coded) and coded ME imaging schemes. For the results shown below, we typically integrate for on the order of 100 seconds for each speckle sub-frame in order to achieve an excellent signal to noise ratio (SNR, typically 100) and overcome the readout detector noise on the CCD camera. We note, however, that the technique does not require this measurement duration or SNR; we find that the performance remains fairly uniform down to approximately 1 s for our system, at which point the image quality of the conventional ME scheme (with a static object and scatterer) is reduced. We note that others have demonstrated conventional ME imaging with integration times as short as 10 ms with alternate sources, configurations, and/or detectors [1], which implies that our technique can be extended to much faster absolute frame rates. Thus, the specific times used in this experiment do not represent a fundamental limit; rather, they demonstrate the potential to realize improved performance relative to conventional ME imaging, and give excellent results for our particular system.

We note that, in order to have a faithful reconstruction in the coded ME imaging, several conditions should be satisfied. Katz et al.[6] have previously discussed five such conditions, which still apply in our technique (as the recovered speckle in each temporal subframe must be in the conventional ME regime). However, our method additional requires that a) the code features should be at least 2x smaller than the speckle structures of interest and b) the coded modulations should be well sampled when imaged into the detector plane.

2. Memory effect angular range

The scatterer used in our experiment is a 600 grit, ground glass diffuser (Thorlabs DG20-600-MD), which is 2 inches in diameter and approximately 2 mm thick. To characterize its memory effect angular range $\Delta\theta_{FOV}$, we shine a collimated λ=630 nm laser (with a diameter of several mm) on the diffuser and record the resulting speckle pattern. We then tilt the diffuser relative to the laser and measure the speckle pattern in 1-degree increments. Supplemental Figure 2a shows examples of the recorded speckle patterns at different relative tilt angles ($\Delta\theta$), from which it is clear that the speckle pattern retains its structure and undergoes a slight shift for small tilt angles. The pattern itself changes at larger $\Delta\theta$, which indicates the maximum angular field of view available for this diffuser. To quantify this limitation, we calculate the maximum value of the cross-correlation between the speckle patterns at a given tilt angle and a reference speckle pattern (measured at $\Delta\theta$=0). This value drops off monotonically as the tilt angle increases (see Supplemental Figure 2b) and is solely due to the change in the structure of the speckle pattern (i.e., it does not reflect changes due to simple shifts). We fit this data with the theoretical memory effect curve, which is given by Feng et al.[2] as $C(\Delta\theta, L) = [k_0\ \Delta\theta\ L/\sinh\ (k_0\ \Delta\theta\ L)]^2$, where C is the maximum cross-correlation, $k_0=2\pi/\lambda$ is the free space wave number, and L is the effective mean free path. While the agreement is not perfect (due to the fact that the diffuser thickness greatly exceeds the mean free path), we find a best-fit for L=1.2 μm, which corresponds to $\Delta\theta_{FOV} \approx \lambda/\pi L = 9.57°$.

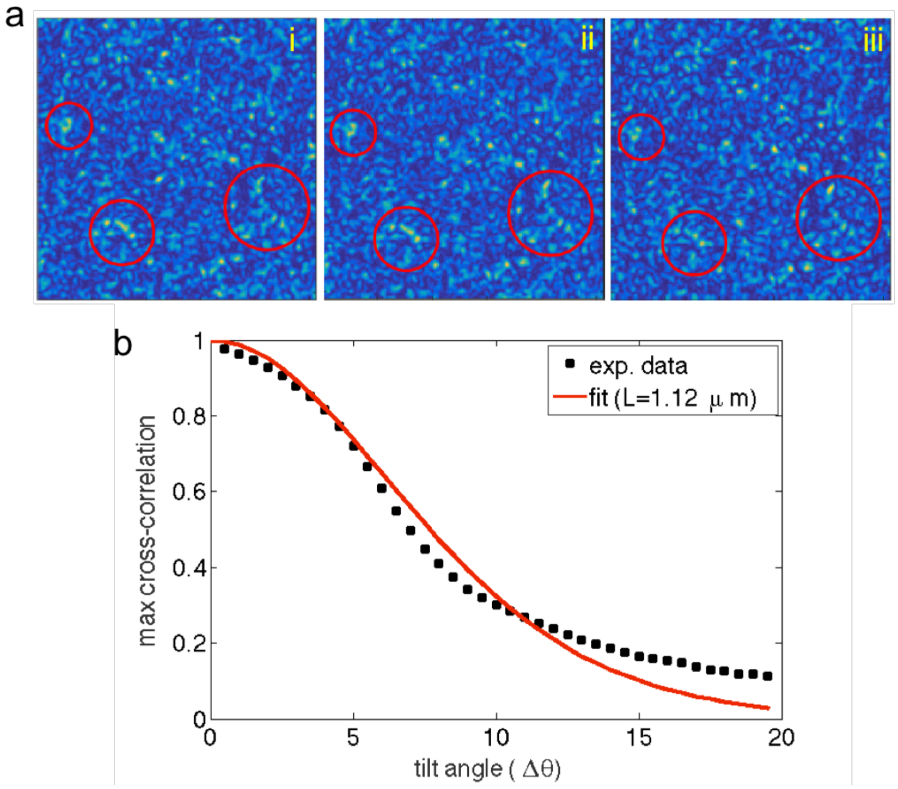

Supplemental Figure 2| Characterization of the angular memory effect for a 600 grit ground glass diffuser: a, Example speckle obtained using a collimated beam with a relative tilt angle of i-iii) 0, 1, and 2 degrees, respectively. The red circles are meant to highlight key landmarks and

guide the eye as the relative tilt angle is varied. **b,** Maximum cross-correlation between the speckle obtained at different tilt angles (solid dots). The red curve is a best fit to the theoretical expected correlation in the diffusive regime with L=1.2 μm[2].

3. Procedure for speckle de-multiplexing

a. Mathematical formulation of the problem

As described by Llull et al.[3], the pixel-wise temporal coding scheme employed here can be formulated in terms of a linear forward model. The coded, multiplexed measurement $I(x,y)$ can be written as

$$I(x,y) = \sum_{i=1}^{N_t} I_i(x,y)\, T_i(x,y),$$

where $I_i(x,y) = I(x,y,t_i)$ is the speckle pattern and $T_i = T(x,y,t_i)$ is the coded aperture pattern produced at time t_i, respectively. Each of these quantities is represented in the discrete pixel basis as an $\mathbb{R}^{n\times m}$ matrix, where n and m are the number of detector pixels in the x and y directions, respectively. Writing the $\mathbb{R}^{nm\times 1}$ vectors

$$Y = [I(1,1), I(2,1), \ldots I(n,m)]^T$$
$$X_i = [I_i(1,1), I_i(2,1), \ldots I_i(n,m)]^T$$
$$M_i = [T_i(1,1), T_i(2,1), \ldots T_i(n,m)]^T,$$

one can define the $\mathbb{R}^{nm\times nm}$ matrix $H_i = \mathrm{diag}\{M_i\}$, where diag{A} is a matrix with the elements of A along the diagonal and *i=1,2,…N*$_t$. Using these definitions, we write the forward model as

$$Y = HX,$$

where $X = [X_1^T, X_2^T, \ldots, X_{N_t}^T]^T$ is an $\mathbb{R}^{N_t nm\times 1}$ vector and $\mathrm{H} = [H_1, H_2, \ldots, H_{N_t}]^T$ is an $\mathbb{R}^{N_t nm\times N_t nm}$ matrix. The problem of reconstructing the speckle sub-images I_i from the compressive measurement can be cast as the optimization problem

$$\hat{X} = \mathrm{argmin} \parallel X \parallel_2 \quad \text{subject to} \parallel HX - Y \parallel_2^2.$$

b. Dictionary learning for speckle representation using BPFA

The optimization problem described above is well-known[4] and solvable to a high accuracy if the underlying images can be sparsely represented via a dictionary. The next step of the speckle recovery processing therefore involves finding an appropriate dictionary. Sparse representation via dictionary is possible, as speckle is not truly random and contains spatial correlations on the scale of the grain size when it is oversampled by the detector. In our experiment, the smallest object is a 0.144×0.144 mm^2 square, and the oversampling of speckle is about 10x. In general, dictionary learning is a method for representing a set of data as a weighted sum of a small number of representative "dictionary elements." For the problem at hand, this corresponds to finding a representation

$$X_i = D\,\alpha_i$$

where $D = [d_1, d_2, \dots d_K]$ is the overcomplete dictionary composed of elements $d_i \in \mathbb{R}^{nm\ x\ 1}$ and $\alpha_i = [\alpha_{i1}, \alpha_{i2}, \dots \alpha_{iK}]$ is the sparse coefficient vector. Here, $K \ll nm$ and only a small number of cofficients should be non-zero.

While various techniques exist for determining the dictionary[4,5], we use a recently developed Bayesian nonparametric method known as beta process factor analysis (BPFA[5]), which is capable of inferring the sparsity level and also the threshold error automatically. In addition, BPFA is specifically designed for operating on compressed measurements, and automatically prunes the unneeded elements and updates the sparsity pattern by using the posterior distribution of a Bernoulli process. To train the dictionary D, we first measure the speckle pattern produced by individual diffraction-limited points at the object plane (i.e. the point spread function, or PSF, of the system). Since all possible speckle patterns are composed of superpositions of such speckle patterns, it is natural to think of the PSF as representing a basis element for the dictionary (see Supplemental Figure 3a). From this 1024 x 1024 pixel speckle image, we chose non-overlapping 16 x 16 pixel patches, which results in 4096 separate patches. We choose this patch so that a single patch only contains a small number of speckle grains. By using 18 different raw (i.e., unprocessed) speckle PSF realizations our final training data corresponds to 73728 different 16 x 16 pixel patches, which can be represented as an $\mathbb{R}^{256 \times 73728}$ matrix.

Once the training data is acquired and dictionary size is specified, the dictionary training process is essentially a Gibbs sampling process[6]. BPFA treats the dictionary elements, weights, indicator and hyperpriors as random variables. The algorithm first draws the initial value of all variables from certain prior distributions, then iteratively updates the posterior distribution for the variables and resamples until the stationary distribution of the dictionary elements converges. The complexity of each iteration is $\mathcal{O}(\mathrm{KBN}^2)$, where K is the number of dictionary elements, B is the dimension of each dictionary element, and N is the amount of training data. For our implementation, we typically use 100 iterations of BPFA, which takes approximately 40 hours to train offline.

Supplemental Figure 3b shows the K=512 dictionary elements determined using BPFA, which is significantly smaller than the 1024 x 1024 = 1,048,576 elements required in the pixel basis. By visual inspection, one can see that the characteristics of the dictionary elements resemble that of the speckle features, code elements, and noise in the raw measurements. We note that this sparse basis is quite different from the commonly-used wavelet basis used in natural imagery; we describe the impact of this basis choice below in Sec. 3d.

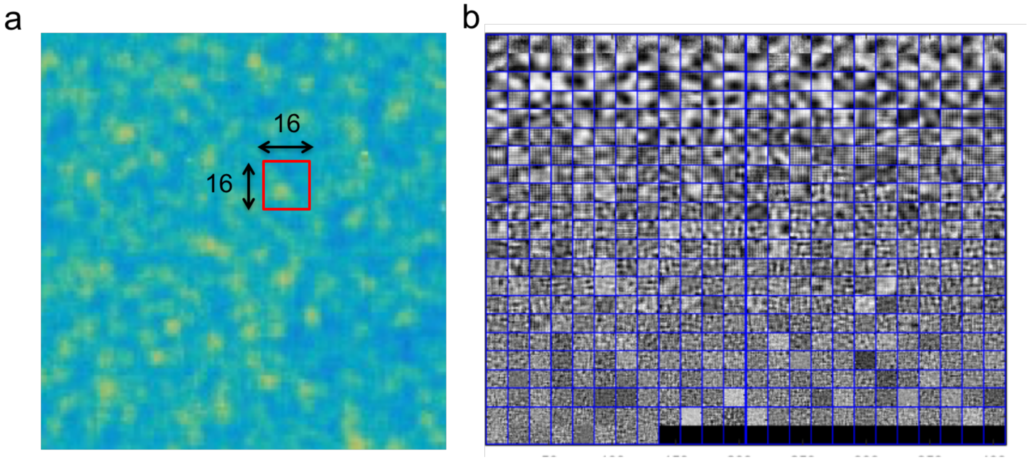

Supplemental Figure 3 | Dictionary represntation of speckle: a, A processed speckle pattern produced by a static, diffrcation-limited object is segmented into 16 x 16 pixel patches to be used as training data. **b,** Visual representation of the 512 learned dictionary elements

The resulting dictionary does an excellent job at representing the speckle patterns of interest. Supplemental Figure 4 a1-d1 show examples of measured speckle patterns for the letter 'U' (as shown in manuscript Fig. 3) along with the associated dictionary-based representation of the speckle (Supplemental Figure 4 a2-d2). While the object remains the same, the speckle pattern is different in each case because of changes to other elements in the system. For example, Supplemental Figure 4 a and b correspond to the experimental system described in the Methods section at an SNR of 18 and 2, respectively. In Supplemental Figure 4 c and d we vary the average speckle size by changing the magnification of the relay optics and the diffuser (we use instead a 200 grit diffuser), respectively. For all cases, the representation results in a correlation between the measurement and dictionary representation of larger than 0.9999 when 80 or fewer dictionary elements are used, which demonstrates the generality of the dictionary. Thus, while training the dictionary requires on the order of 10s of hours, it is done off-line and needs to be done only once.

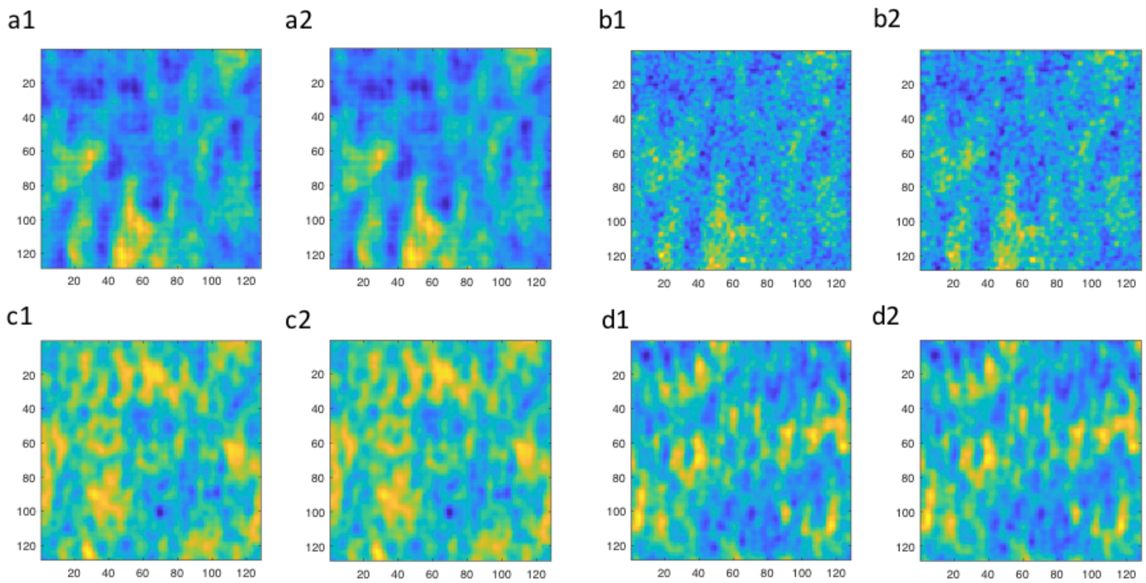

Supplemental Figure 4 | Generality of the speckle dictionary: a-d) Speckle images from the setup described in the Methods section at an SNR of 18 and 2, obtained by varying the diffuser-to-detector distance and relay optics magnification to alter the average speckle size, and using a different (200 grit) diffuser, respectively. **a1-d1** Show the measured speckle and **a2-d2** show a representation of the speckle using the dictionary, respectively.

c. Sub-frame speckle reconstruction via OMP

Using a dictionary representation of the speckle patterns allows us to reformulate the forward model described above as

$$Y = HD_c\alpha \ ,$$

where the full $D_c = \text{blockdiag}\{D, D, \dots D\}$ is a block diagonal $\mathbb{R}^{N_t nm \times N_t K}$ matrix because all speckle patterns can be represented by the same dictionary, and $\alpha \in \mathbb{R}^{N_t nm \; x \; 1}$ is the vector of sparse coefficients representing each speckle pattern. The optimization problem is therefore reduced to determining the optimally sparse coefficients given the known dictionary D according to

$$\hat{\alpha} = \text{argmin} \parallel \alpha \parallel_2 \quad \text{subject to} \parallel HD_c\alpha - Y \parallel_2^2.$$

This problem can be solved using the orthogonal matching pursuit (OMP) algorithm[7] using a given sparsity level L (we typically choose L=80 as a compromise yielding high-quality estimated speckle in a reasonable runtime). Psuedocode for the OMP recovery algorithm is shown in Supplemental Table 1. While we implement this algorithm in Matlab on a CPU (as described in the Methods section), speedups on the order of 10-100 are possible through adaptively choosing the sparsity level, implementing more sophisticated stopping criteria, and/or compiling and running on a GPU[8].

Algorithm 1 OMP for signal recovery

1: Initialize the residual $r_0 = y$, the index set $\Gamma_0 = \varnothing$, an empty matrix Φ_0, and iteration number $t = 1$.

2: Find the index λ_t that solves the simple optimization problem:

$$\lambda_t = \mathrm{arg}max_{j=1,2,\ldots,d} \| < r_{t-1}, A_j > \|.$$

3: Update $\Gamma_t = \Gamma_{t-1} \cup \{\lambda_t\}$ and $\Phi_t = [\Phi_{t-1}\ A_{\lambda_t}]$.

4: Solve a least square estimation problem to obtain a new estimate of x(can use pseudo inverse):

$$x_t = \mathrm{arg}min_x \|y - \Phi_t x\|_2.$$

5: Calculate the new residual:

$$r_t = y - \Phi_t x_t.$$

6: repeat the process till $t = L$ or $\|r_t\| < \epsilon$.

Supplemental Table 1 | Pseudocode for the OMP algorithm

d. Performance bounds and impact of recovery algorithm

We consider how the quality of the recovered images depends on the degree of multiplexing. Supplemental Figure 5a shows the fidelity of the recovered speckle as a function of the number of reconstructed frames obtained from a single, coded measurement N_t, which can also be interpreted as the degree of compression (i.e., ratio of the number of recovered to measured pixels in the raw speckle image). To quantify the performance, we compute the correlation between the speckle measured using conventional ME imaging (i.e., with all elements static) and recovered using our coded ME imaging approach (i.e., with full dynamics). From this, we see that the quality of the speckle decreases monotonically with increasing N_t. To clarify the relationship between our correlation metric and image quality, Supplemental Figure 5b shows the first frame of the estimated object for different values of N_t (the recovered speckle quality is largely uniform across subframes). The image quality degrades slowly with N_t, and we find that the object is still recognizable after $N_t = 8$ (corresponding to a correlation of 0.73).

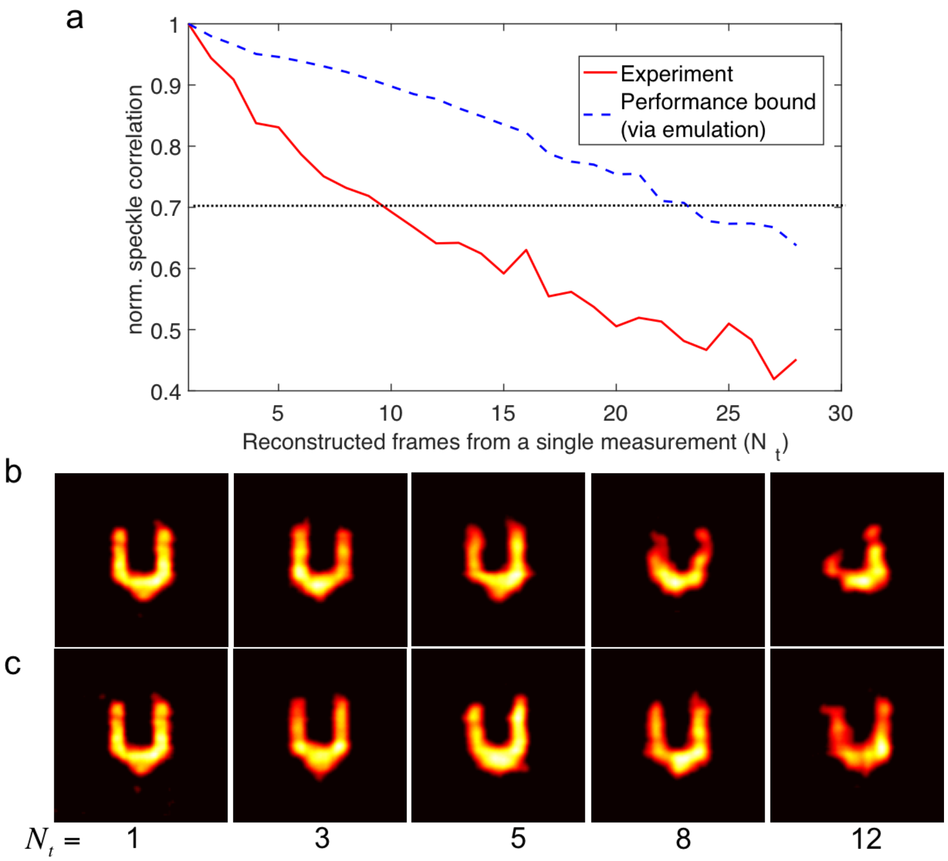

Supplemental Figure 5 | System performance for different levels of multiplexing. a, Normalized correlation between the speckle obtained using multi-shot, conventional and coded, single-shot ME imaging as a function of the number of recovered frames per detector measurement. The object is first four objects are the letters 'D', 'U', 'K', 'E' and, for $\boldsymbol{N_t} > \mathbf{4}$, we include additional similar objects. The solid red and dashed blue curves represent the performance of the experimental and emulated systems, respectively. **(b-c),** First frame of the estimated sequence for different values of $\boldsymbol{N_t}$ obtained using the experimental and emulated systems, respectively.

While we experimentally demonstrate nearly an order of magnitude frame rate improvement, we find that imperfect characterization of the imposed code pattern, due to imaging artifacts and noise, limit the performance of our experimental system. To assess the potential performance at a more fundamental level, we emulate coded data by using the multi-shot, conventional ME approach to obtain uncoded speckle images, imposing an ideal digital modulation on each image, and summing the artificially coded speckle images. We find that we can recover nearly three times as many frames as in the experiment with a similar image degradation (see Supplemental Figure 5c). While this represents a step toward quantifying the ultimate performance limit, we note that a rigorous analysis of the system is challenging because of the nonlinear nature of the involved estimation algorithms. However, one can optimize performance by making maximal use of the detector dynamic range. In addition, using smaller code features (limited by the detector pixel size)

and a larger detector area allow one to capture more coded speckles, which improves the performance of both the demultiplexing and phase retrieval algorithms.

The choice of demultiplexing algorithm (including the associated priors and choice of sparse representation) also impacts the system performance. Supplemental Figure 6 shows the fidelity of the recovered speckle obtained using the Generalized Alternating Projection (GAP[9]) and OMP with BPFA algorithms. GAP uses a wavelet basis as a sparse representation of the speckle; however, speckle is not a natural image and the performance is correspondingly limited. As a dictionary learning approach that accurately reflects the structure of the speckle pattern, BPFA combined with OMP results in significantly improved performance in both the experimental and emulated (indicating an upper bound performance limit) data. This improvement is particularly significant at large N_t where the degree of multiplexing is increased and the SNR is reduced. This further confirms our observation that speckle is not truly random (as it is typically considered to be); rather, the correlations present in the speckle contain information, and a representation that takes these correlations into account allows us to best recover and/or preserve this information.

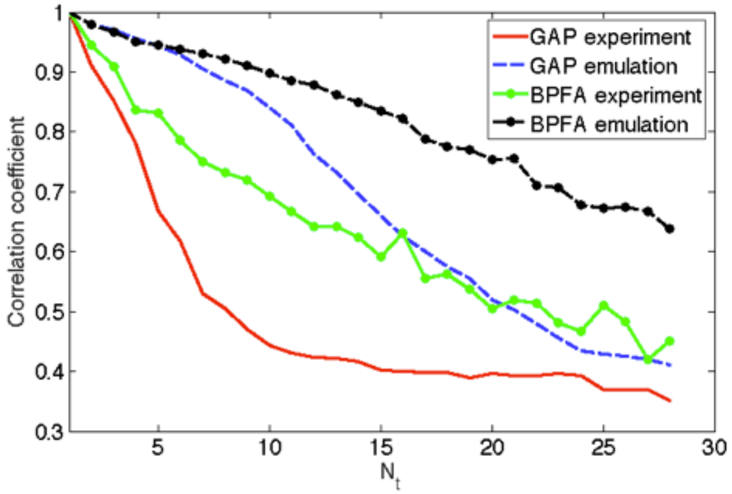

Supplemental Figure 6 | System performance for different levels of multiplexing: Normalized correlation between the speckle obtained using multi-shot, conventional and coded, single-shot ME imaging as a function of the number of recovered frames per detector measurement. The object is first four objects are the letters 'D', 'U', 'K', 'E' and, for $\mathbf{N_t} > \mathbf{4}$, we include additional similar objects. The solid red (green) and dashed blue (black) curves represent the performance of the experimental and emulated systems, respectively, using the GAP (OMP with BPFA).

e. Impact of effective object complexity

As discussed by Katz et al.[1], the performance of the conventional ME imaging system depends on the object itself. In particular, Katz et al. show that the object complexity N, which is defined as the number of bright, diffraction-limited resolution cells in the object during a single measurement, must be less than the camera full depth. Said another way, the object sparsity must be such that the speckle contrast exceeds a certain SNR (which, in turn, is determined by the choice of phase retrieval algorithm). While the object complexity was defined with respect to static objects, it generalizes directly to time-dependent objects such that the overall complexity depends both on the shape and dynamics of the object. Thus, it can be immediately seen that excessive object motion over the time

scale of a single measurement acquisition, even in the case of objects that are sparse in the spatial domain, can compromise the performance of the conventional ME imaging system. While Katz et al. state that these limitations can be circumvented by acquiring multiple speckle measurements in series and averaging the resulting autocorrelations, this approach is not possible for objects whose dynamics are less than or even on the order of the measurement time. By boosting the frame rate and/or measurement SNR, our coded approach reduces the effective object complexity within a given subframe and extends the range over which ME imaging can be implemented.

f. Emulation of continuous moving diffuser and temporally mismatched coding

While the results shown in Figs. 3-5 involve discrete diffuser and/or object dynamics, our coding technique is not limited to such dynamics. Supplemental Figure 7a shows the emulated case of a static object imaged through a moving scatterer. The object is the same letter 'L' shown in Fig. 4, but the diffuser's linear translational motion is now quasi-continuous in the horizontal direction. More specifically, the diffuser moves through 48 different locations (each separated by an increment of only 120 μm) within a single detector acquisition time. For the case of conventional ME imaging, the static object appears blurred by the diffuser motion (see the associated autocorrelation and recovered image in Supplemental Figure 7 a). However, our coded aperture technique allows us to mitigate this blur and recover a crisp image of the static object. Supplemental Figure 7 b and c shows a single, representative result for a single subframe recovered with N_t=4 and 12 (although we note that 4 and 12 different images are produced), respectively. As expected, shorter sub-frames (i.e., larger values of N_t) reduce the blurring caused by the moving scatterer, despite the fact that we have in no way assumed that the object is static throughout the acquisition time.

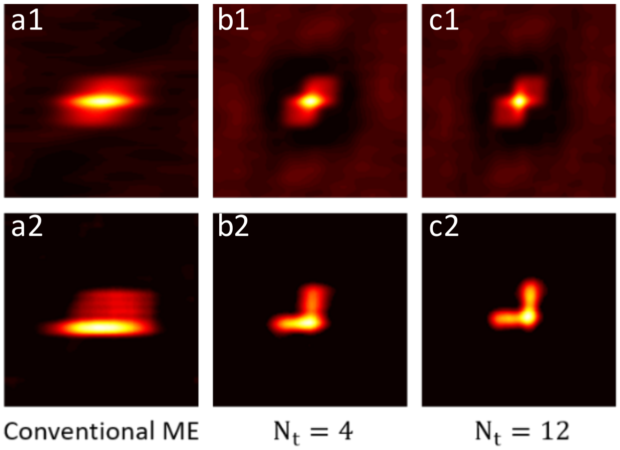

Supplemental Figure 7 | Emulation of a quasi-continuously moving diffuser: a1-c3) The autocorrelation and a2-c2) recovered image for the case of a static object and a scatterer undergoing quasi-continuous linear translation motion when $\boldsymbol{N_t}$=1 (conventional ME), 4, and 12, respectively. The observed blur of the object (the letter 'L') is reduced as $\boldsymbol{N_t}$ increases.

As a second and related scenario, we consider the case of a static diffuser and dynamic object. The scenario is identical to the 'D' and 'K' letters shown in Fig. 3 except that we now assume that the coded aperture timing is not synchronized exactly with the object dynamics. More specifically, we consider the case where the object present during a particular temporal subframe (as defined by the time over which the speckle image is modulated by a single code pattern) is composed of a linear superposition of two temporally adjacent objects. The resulting speckle image is therefore described by $I(x,y) = r\,I_D(x,y) + (1-r)\,I_K(x,y)$, where r is the temporal overlap of the first object within the temporal subframe and I_K and I_D are the speckle patterns resulting from the letter 'K' and 'D', respectively. Supplemental Fig. 8 shows that, for small (large) r, the resulting image is dominated by the letter 'K' ('D'). For intermediate values of r, the resulting image shows is a weighted sum of the overlapping objects (as confirmed by comparing the appropriate columns of Supplemental Figure 8 b and c). Thus, we find that our method is robust to the absolute timing of the system dynamics relative to the imposed coding.

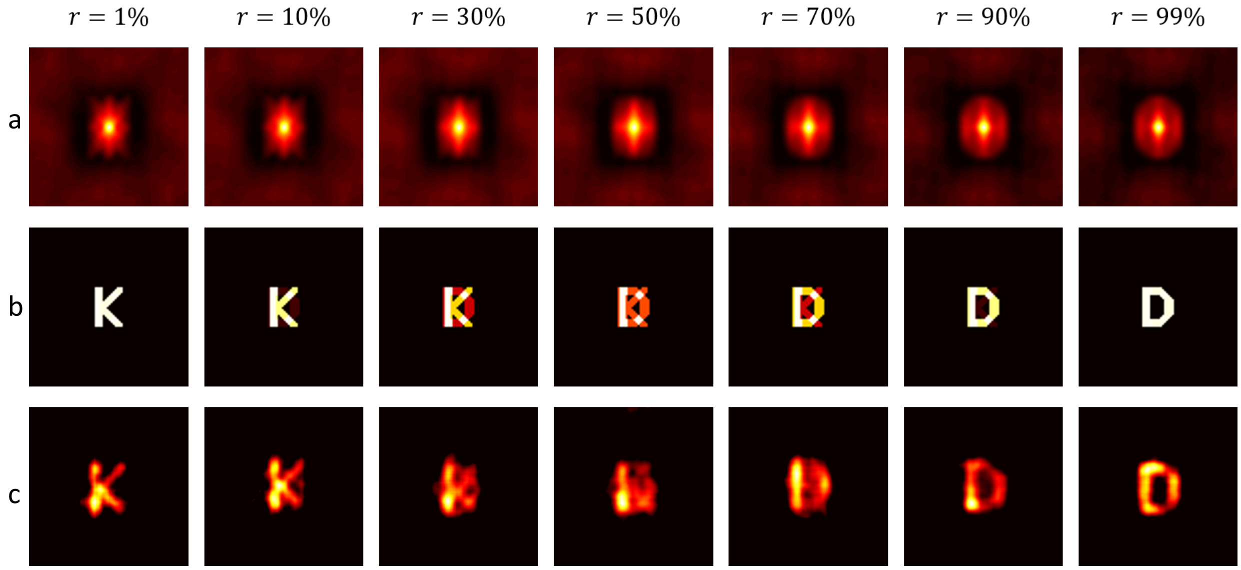

Supplemental Figure 8 | Emulation of temporally mismatched coding a) autocorrelation b) ground truth linearly combined object and c) recovered image for the case of a dynamic object that evolves discretely between the letter 'D' and 'K' with varying degrees of temporal overlap, r, within a subframe. We assume that the scatterer is static, and that the object starts out as the letter 'D' and changes to the letter 'K' while a single code pattern is used to modulate the scene. The resulting image resembles a weighted superposition of the two objects.

To guarantee high-fidelity images, one therefore needs to make sure that the coding rate is comparable to the object and/or scatterer dynamics. Assuming sufficiently bright illumination, the coding rate is limited by the coding device refresh rate. For applications where an SLM disallows sufficiently fast coding, one can choose a DMD or a moving (translating or rotating) physical coded aperture.

g. Impact of code feature size

We next consider the impact of the code feature size on image quality. The key physical length scale in the system is the average speckle grain size; we therefore study the dependence on code

feature size relative to the speckle size by performing an emulation study in which we used un-coded, experimentally-measured speckle and digitally-imposed code patterns with different feature sizes. We start by acquiring speckle for a small, square object with an extent of 144 μm x 144 μm, which generates speckle with an average grain size of 10 pixels on the coding SLM. As shown in Supplemental Fig. 9, we need a code feature size of 5 pixels or less (in each dimension) to recover a high-fidelity representation of the speckle (as quantified by the correlation between the ground truth and recovered speckle patterns, as in Supplemental Fig. 5). Thus, we find that we need approximately Nyquist coding within a speckle to accurately recover each grain. Interestingly, if we repeat this experiment with a nearly 4x larger object (here we use the letter 'L', which produces roughly 4x larger speckle grains), we find that the maximum allowable code feature size is still about 5 pixels. We understand this result by noting that, while the total extent of this object is larger than the initial one, the smallest structure in the 'L' is comparable in size to the small square object (i.e. the vertical and horizontal components of the 'L' are approximately 144 μm in size). Thus, we find that high-quality image recover requires on the order of 2 x 2 coding applied to the smallest spatial scale of interest in the object.

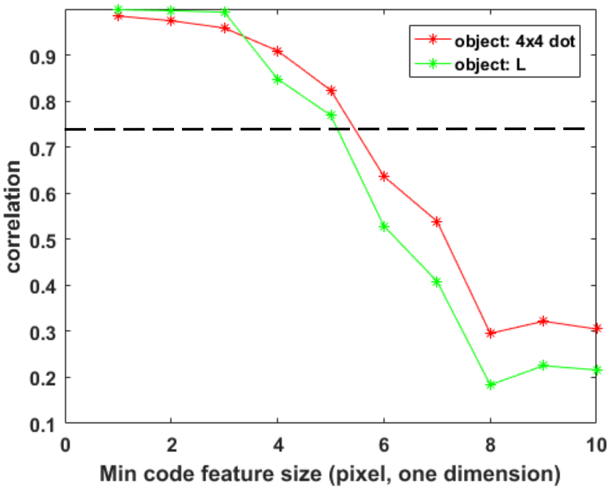

Supplemental Figure 9 | Impact of code feature size: Correlation between the recovered and ground truth speckle as a function of the size of the coded aperture features (in units of pixels along a single direction). The red (green) curve shows how the fidelity of the recovered speckle decreases as the code feature size increases for a small square object (larger object in the shape of an 'L'). Based on Supplemental Figure 5, we define the cutoff at a correlation value of 0.73, at which point the recovered image quality degrades noticeably.

h. Advantage of the compressed-sensing/dictionary learning scheme

In this section, we focus on demonstrating the value of using a non-linear, compressed-sensing algorithm for speckle recover as compared to a linear algorithm. While most conventional linear coding approaches involve making multiple, separate measurements with different codes to realize a multiplexing advantage over detector noise, we want to focus on a direct comparison in which the imaging is done in a single measurement (i.e., snapshot). We therefore introduce a coding method that can be described as a sort of temporal Bayer filter, in which spatial resolution is traded for enhanced temporal resolution. In the simplest example of this scheme, consider a macro-pixel

coding scheme composed of a 2x2 block. This method allows one to realize the case of $N_t = 4$ temporal subframes by opening one pixel in each macro pixel in series during a single acquisition (see Supplemental Fig. 10 a). The speckle is then recovered by cubic interpolation (i.e. a linear algorithm) of the blocked regions at each temporal sub-frame, and the result is then convolved with a 7x7 pixel kernel to remove pixilation artifacts and improve the image quality. The resulting average correlation between the ground truth and recovered speckle for this scenario is 0.94 (without convolution with the blur kernel, we find a correlation of only 0.86, see Supplemental Fig. 10 b1). Using the same coding strategy in combination with our dictionary-learning/compressed sensing algorithm, we find instead an average correlation of 0.98, as shown in Supplemental Fig. 10 b2. In fact, our compressive coding approach (using a random code pattern) can operate at $N_t = 8$ before the recovered speckle fidelity drops to 0.94 (the quality of the linear recovery scheme with $N_t = 4$). Thus, we find that our approach offers the advantage of superior image quality for fixed N_t or, alternatively, larger values of achievable N_t for the same image quality. In addition (and not considered above), any linear, single-shot scheme can only measure 1/N of the available light in each temporal sub-frame; in contrast, our compressive approach measures up to ½ of the light. We therefore also expect significant SNR benefits, relative to a linear measurement approach.

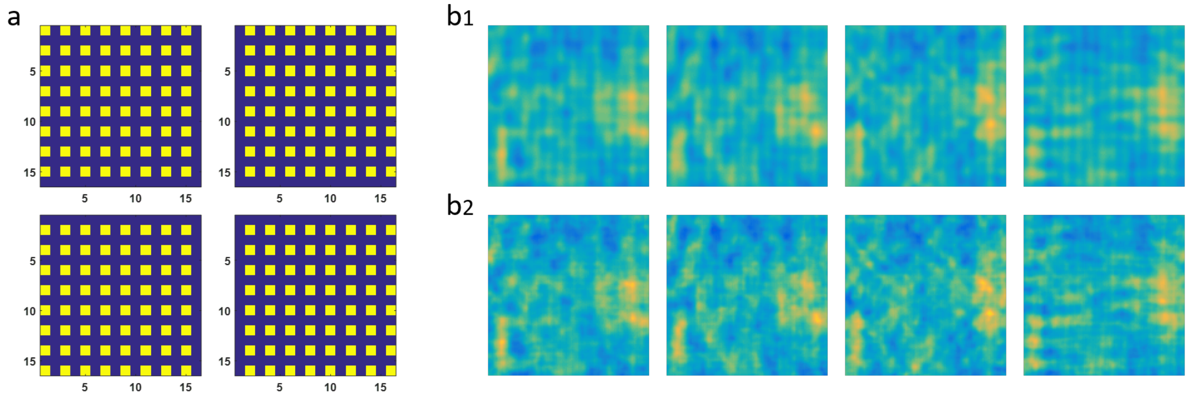

Supplemental Figure 10 | Advantage of nonlinear recovery algorithms: a) illustration of the single-shot coding scheme that enables linear processing of the resulting multiplexed speckle image. Each image represents the imposed code at different times, where we use a 2x2 macro-pixel to realize N_t=4 subframes. b1-2) Four patches of speckle sub-frames recovered using the linear processing approach and compressive/dictionary-learning processing approach. The average correlation with the ground truth is 0.94 and 0.98, respectively.

4. Object recovery via phase retrieval (gradient descent)

After demultiplexing the speckle, we calculate the autocorrelation of each frame separately. As described by Katz et al.[1], one can recover the object from the autocorrelation using a phase retrieval algorithm. The autocorrelation pattern, $R(x,y)$ is calculated using the processed speckle image $\hat{I}(x,y)$:

$$R(x,y) = \hat{I}(x,y) \star \hat{I}(x,y) = FT^{-1}\{\left|FT\{\hat{I}(x,y)\}\right|^2\}.$$

The power spectrum A_F, which is the amplitude of the object in the Fourier domain, is obtained by inverse Fourier transforming the autocorrelation $R(x,y)$:

$$A_F(k_x, k_y) = \sqrt{|FT^{-1}\{R(x,y)W(x,y)\}|}\,,$$

where $W(x,y)$ is a 2D Tukey window function chosen to mitigate edge effects.
We use the conjugate gradient descent (CGD) phase retrieval algorithm,[13] which converges faster than the classical error reduction and hybrid input output algorithms. The flow chart of the algorithm is presented in Supplemental Figure . The initial guess in the object domain $g_0(x,y)$ is randomly selected. In the kth iteration, the algorithm updates the real and imaginary parts of the estimate according to:

(1) $G_k(k_x, k_y) = FT\{g_k(x,y)\}$,
(2) $\theta_k(k_x, k_y) = arg\{G_k(k_x, k_y)\}$,
(3) $G_k'(k_x, k_y) = A_F(k_x, k_y)e^{i\theta_k(k_x,k_y)}$,
(4) $g_k'(x,y) = FT^{-1}\{G_k'(k_x, k_y)\}$,
(5) $g_k''(x,y) = g_k'(x,y) + h[g_k'(x,y) - g_{k-1}'(x,y)]$,
(6) $g_{k+1}(x,y) = \begin{Bmatrix} 0, & \text{for } (x,y) \in \Gamma \\ g_k''(x,y), & \text{otherwise} \end{Bmatrix}.$

In step (5), h is the step size parameter that takes g_{k+1} closer to the optimal solution from g_k'' than from g_k'. The step size controls the convergence of the algorithm. In our application, $h = 0.18$ (we note that, when $h = 0$, CGD becomes standard error-reduction algorithm). Also, in step (6), Γ is the set of pixels that violate the "non-negativity and realness" constraints. For our system, we typically use 3600 iterations per run and perform the phase retrieval with different initial conditions between 10-100 times.

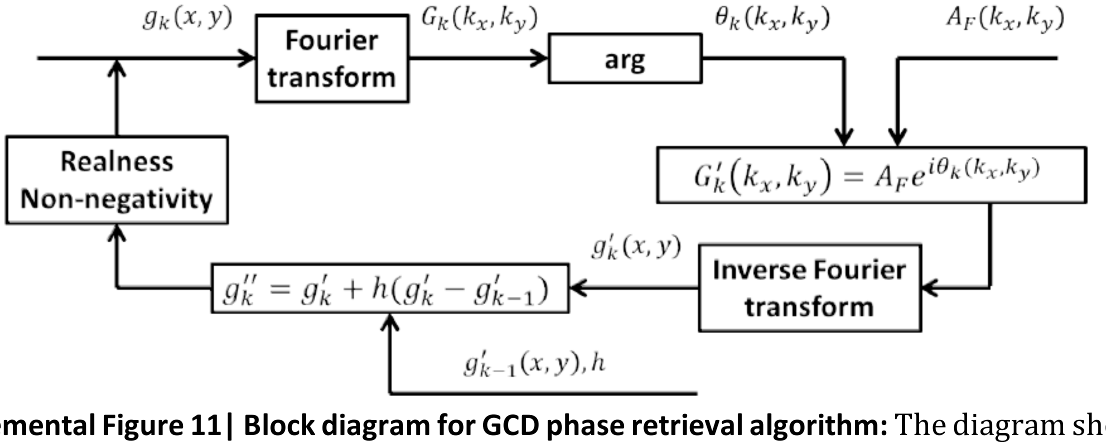

Supplemental Figure 11| Block diagram for GCD phase retrieval algorithm: The diagram shows that the GCD algorithm is a modified version of Gerchberg-Saxton algorithm. $\mathbf{A_F}(\boldsymbol{k_x}, \boldsymbol{k_y})$ is the Fourier amplitude which is obtained from the experimental measurement. Compared with the HIO phase retrieval algorithm implemented by both Katz et al.[1] and Bertolotti et al.[14], the main difference is that the GCD applies the phase retrieval result from last iteration to speed up the convergence procedure.

References:

1 Katz, O., Heidmann, P., Fink, M. & Gigan, S. Non-invasive single-shot imaging through scattering layers and around corners via speckle correlations. *Nat Photon* **8**, 784-790, doi:10.1038/nphoton.2014.189 (2014).

2 Feng, S., Kane, C., Lee, P. A. & Stone, A. D. Correlations and fluctuations of coherent wave transmission through disordered media. *Physical review letters* **61**, 834-837, doi:10.1103/PhysRevLett.61.834 (1988).

3 Llull, P. *et al.* Coded aperture compressive temporal imaging. *Optics express* **21**, 10526-10545, doi:10.1364/OE.21.010526 (2013).

4 Chen, H. W., Kang, L. W. & Lu, C. S. in *28th Picture Coding Symposium.* 210-213.

5 Zhou, M. *et al.* Nonparametric Bayesian Dictionary Learning for Analysis of Noisy and Incomplete Images. *IEEE Transactions on Image Processing* **21**, 130-144, doi:10.1109/TIP.2011.2160072 (2012).

6 Stuart, G. & Donald, G. Stochastic Relaxation, Gibbs Distributions, and the Bayesian Restoration of Images. *IEEE Trans. Pattern Anal. Mach. Intell.* **6**, 721-741, doi:10.1109/tpami.1984.4767596 (1984).

7 Cai, T. T. & Wang, L. Orthogonal Matching Pursuit for Sparse Signal Recovery With Noise. *IEEE Transactions on Information Theory* **57**, 4680-4688, doi:10.1109/TIT.2011.2146090 (2011).

8 Fang, Y., Chen, L., Wu, J. & Huang, B. in *2011 IEEE 17th International Conference on Parallel and Distributed Systems.* 1044-1047.

9 Xuejun, L., Hui, L. & Lawrence, C. Generalized Alternating Projection for Weighted-$\ell_{2,1}$ Minimization with Applications to Model-Based Compressive Sensing. *SIAM Journal on Imaging Sciences* **7**, 797-823, doi:10.1137/130936658 (2014).

10 Fellgett, P. B. On the Ultimate Sensitivity and Practical Performance of Radiation Detectors. *J. Opt. Soc. Am.* **39**, 970-976, doi:10.1364/JOSA.39.000970 (1949).

11 Bialkowski, S. E. Overcoming the Multiplex Disadvantage by Using Maximum-Likelihood Inversion. *Applied Spectroscopy* **52**, 591-598, doi:10.1366/0003702981943923 (1998).

12 Sellar, R. G. & Boreman, G. D. Comparison of relative signal-to-noise ratios of different classes of imaging spectrometer. *Appl. Opt.* **44**, 1614-1624, doi:10.1364/AO.44.001614 (2005).

13 Fienup, J. R. Phase retrieval algorithms: a comparison. *Appl. Opt.* **21**, 2758-2769, doi:10.1364/AO.21.002758 (1982).

14 Bertolotti, J. *et al.* Non-invasive imaging through opaque scattering layers. *Nature* **491**, 232-234, doi:10.1038/nature11578 (2012).
